# 2025 ESC/EACTS valvular heart disease guidelines: practical updates on mitral and tricuspid regurgitation

**DOI:** 10.1093/eurheartjsupp/suag001

**Published:** 2026-03-09

**Authors:** Marianna Adamo, Mauro Massussi, Nina Ajmone Marsan, Michael A Borger, Fabien Praz, Marco Metra

**Affiliations:** Department of Medical and Surgical Specialties, Radiological Sciences, and Public Health, University of Brescia, Piazza del Mercato 15, Brescia 25121, Italy; Cardiac Catheterization Laboratory and Cardiology, ASST Spedali Civili di Brescia, Piazzale Spedali Civili 1, Brescia 25123, Italy; Cardiac Catheterization Laboratory and Cardiology, ASST Spedali Civili di Brescia, Piazzale Spedali Civili 1, Brescia 25123, Italy; Department of Cardiology, Leiden University Medical Center, Albinusdreef 2, Leiden 2300 RC, The Netherlands; University Clinic of Cardiac Surgery, Leipzig Heart Center, Leipzig, Germany; Inselspital Universitätsspital Bern, Bern, Switzerland; Vita—Salute San Raffaele University, Milan, Italy; Department of Cardiology, IRCCS San Raffaele Scientific Institute, Milan, Italy

**Keywords:** Clinical practice guidelines, Valvular heart disease, Mitral regurgitation, Tricuspid regurgitation, Transcatheter valve intervention

## Abstract

2025 ESC/EACTS guidelines for valvular heart disease reflect the rapid evolution of transcatheter technologies and multidisciplinary care. In primary mitral regurgitation, surgical repair remains the gold standard, with a new Class I recommendation for asymptomatic patients and refined timing incorporating indexed left ventricular end-systolic dimension. Transcatheter edge-to-edge repair (TEER) is upgraded to Class IIa for symptomatic high-risk patients. In secondary mitral regurgitation (SMR), the guidelines distinguish between atrial and ventricular phenotypes. Transcatheter edge-to-edge repair has now a Class I [Level of Evidence (LOE) A] recommendation for very selected patients with ventricular SMR symptomatic despite guideline-directed medical therapy. For atrial SMR, surgery—often involving concomitant atrial fibrillation ablation—is preferred in suitable candidates, while TEER is reserved to high-risk patients. Regarding tricuspid regurgitation, the guidelines emphasize early referral and structured evaluation of right ventricular function and pulmonary pressures (Class I). Transcatheter tricuspid valve interventions are upgraded to Class IIa (LOE A) for symptomatic high-risk patients, provided severe right ventricular dysfunction or precapillary pulmonary hypertension are absent. These updates redefine atrio-ventricular valve management by promoting earlier and personalized intervention. They solidify the role of transcatheter therapies as evidence-based, outcome-modifying options within a Heart Valve Centre framework, while precisely delineating the continued priority of surgery in lower-risk populations and complex anatomies.

## Introduction

Evidence supporting the treatment of atrioventricular valves in presence of severe regurgitation are growing. Several randomized controlled trials as well as observational studies have been published in the last years, leading to important updates in the recommendations reported in the recent 2025 ESC/EACTS guidelines for the management of valvular heart disease.

The aim of this review is to: (i) summarize novel messages reported in the 2025 ESC/EACTS guidelines for the management of valvular heart disease on diagnostic work-up and indications for treatment of mitral regurgitation (MR) and tricuspid regurgitation (TR); and (ii) to report evidence that new and updated recommendations are based on.

## Primary mitral regurgitation

Primary mitral regurgitation (PMR) is characterized by intrinsic disease of the mitral valve leaflets, chordae, papillary muscles, and/or annulus. Surgical mitral valve repair remains the treatment of choice whenever durable results are expected, as it provides excellent long-term survival and avoids prosthesis-related complications.^1^

**Figure 1:**
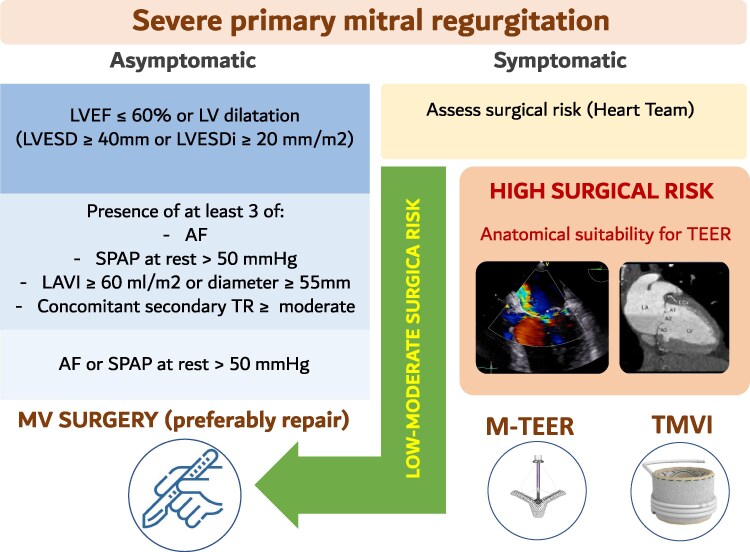
Management algorithm for primary mitral regurgitation. Adapted from Praz *et al*.^2^

A major novelty in the 2025 ESC/EACTS guidelines is the refinement of Class I recommendations for surgery in asymptomatic patients.^2^ Compared with the 2021 version, the current document explicitly incorporates the indexed left ventricular end-systolic dimension (LVESDi) into decision-making and reports 4 specific additional criteria for patients with preserved left ventricular (LV) performance. The addition of LVESDi is a subtle but clinically important change, improving risk stratification in younger or smaller patients, in whom absolute LVESD may underestimate early LV remodelling. Also, the incremental role of other echocardiographic beyond the left ventricle is a crucial update (*Figure 1*).

Another relevant update is the upgrading of transcatheter edge-to-edge repair (TEER) for PMR. While in the 2021 guidelines TEER carried only a Class IIb, LOE B recommendation, in 2025 it has been promoted to Class IIa, LOE B.^3^

### Diagnostic assessment

The 2025 ESC/EACTS guidelines confirm transthoracic echocardiography (TTE) as the cornerstone for the evaluation of PMR, but they place stronger emphasis on a comprehensive and multiparametric approach. Beyond identifying lesion type (prolapse, flail, calcification, mitral annular disjunction), quantification of regurgitation severity with effective regurgitant orifice area (EROA), regurgitant volume, and regurgitant fraction is highlighted for its prognostic impact.^4^

Importantly, the new guidelines stress the role of transoesophageal echocardiography (TOE), particularly three-dimensional TOE, not only for surgical planning but also for selection of patients suitable for transcatheter interventions. In addition, exercise echocardiography is increasingly recommended for patients with discordant symptoms or borderline haemodynamics, as it may unmask dynamic MR or exercise-induced pulmonary hypertension (PH).^5^

Another notable point is the recommendation for right heart catheterization in selected patients, especially when PH or comorbid lung disease may confound non-invasive estimates.^6^ The guidelines also acknowledge the potential of biomarkers such as N-terminal prohormone of brain natriuretic peptide, which, although non-specific, provide useful information about symptom burden and prognosis.^7^

Taken together, the diagnostic chapter of the new guidelines underscores that timely referral and accurate characterization of MR severity and consequences should go beyond resting TTE, integrating advanced imaging, stress testing, and invasive haemodynamics when appropriate.

### Indications for intervention

2025 ESC/EACTS guidelines reinforces surgical mitral repair as the gold standard for chronic severe PMR when durable results are anticipated, with operative mortality <1% in high-volume centres.^8^ The novelty lies in the expanded emphasis on early surgery: the guidelines explicitly include LVESDi ≥20 mm/m^2^ as a trigger for intervention, together with LVESD ≥40 mm or LVEF ≤60%.^9^ This refinement helps to avoid underestimating remodelling in women and smaller patients, who may otherwise present late with irreversible dysfunction.^10^

Additional adverse prognostic markers—such as AF, systolic pulmonary artery pressure >50 mmHg, left atrial enlargement, and concomitant secondary TR—have been found to be associated with poor outcome even when LV function appears preserved, thus included as key criteria for selection of asymptomatic patients for intervention.^11^

The upgrading of TEER from Class IIb to Class IIa (LOE B) reflects accumulating evidence from contemporary registries showing high procedural success, durable MR reduction, and meaningful quality-of-life improvement in high-risk symptomatic PMR (*Table 1*).^12,13^ Importantly, TEER is no longer framed as a ‘last resort’, but as a validated alternative in anatomically suitable patients who are not candidates for surgery. The future role of TEER in lower-risk populations is currently being defined by two landmark randomized controlled trials (RCTs)s: REPAIR MR (NCT04198870), which is evaluating the non-inferiority of TEER against surgery in patients at moderate surgical risk,^14^ and the NIH-funded PRIMARY MR trial (NCT05051033), which aims to determine if surgical repair maintains its superiority over TEER in a younger (≥65) cohort. These results will be pivotal in determining whether TEER can be offered to a broader spectrum of patients with PMR who are currently directed towards surgery.

**Table 1:** **Key recent randomized controlled trials on interventions in** primary mitral regurgitation, secondary mitral regurgitation**, and** tricuspid regurgitation

Study	Population/number of patients (key inclusion and exclusion)	Intervention and control	Relevant outcomes	Key findings and task force interpretation
Primary mitral regurgitation				
Lim DS, 2022, CLASP IID, PMID:36121247	180 patients with severe symptomatic PMR (3+/4+) at prohibitive surgical risk. Inclusion: central adjudication of PMR; Exclusion: non-PMR, non-anatomical suitability, acceptable surgical risk.	PASCAL system vs. MitraClip system, randomized 2:1.	Primary safety: 30-day major adverse events. Primary effectiveness: MR ≤2 + at 6 months. Secondary: MR ≤1+, functional status, Quality of Life (QoL).	Both systems were safe and effective. Non-inferiority achieved for primary safety (3.4 vs. 4.8%) and effectiveness (96.5 vs. 96.8%). Durability of MR ≤1 + was higher with PASCAL at 6 months (83.7 vs. 71.2%). Task Force (TF) interpretation: expands evidence base for TEER, confirming PASCAL as a valid alternative to MitraClip in prohibitive-risk PMR, supporting the guideline upgrade of TEER to IIa, LOE B in PMR.
Akowuah EF, 2023, UK MINI MITRAL, PMID:37314276	330 patients with PMR undergoing surgical repair in 10 UK tertiary centres. Inclusion: candidates for elective repair by expert surgeons; Exclusion: patients requiring replacement or unsuitable for minimally invasive approach.	Thoracoscopically-guided minithoracotomy repair vs. sternotomy repair, randomized 1:1.	Primary: change in SF-36 physical function score at 12 weeks. Secondary: recurrent MR, QoL, repair durability, safety (death, repeat surgery, HF hospitalization) at 1 year.	No superiority of minithoracotomy for recovery of physical function at 12 weeks (Δ + 0.68 points). Repair rates ≈96% in both groups; MR ≤ mild in 92% at 1 year. Safety outcomes similar (5.4 vs. 6.1%). TF interpretation: minimally invasive and sternotomy approaches are equivalent in terms of repair quality and safety; choice of approach should be individualized based on patient preference, surgeon expertise, and centre experience.
**Secondary mitral regurgitation**				
Stone GW, 2023, COAPT 5-year, PMID:37212443	614 patients with HF (NYHA II–IV) and moderate-to-severe or severe SMR despite maximally tolerated GDMT, randomized at 78 sites in US/Canada. Excluded: severe LV dilatation/dysfunction, severe RV dysfunction, PH, or unsuitable anatomy.	TEER (MitraClip) + GDMT vs. GDMT alone.	Primary: HF hospitalizations at 2 years. Extended follow-up: annualized HF hospitalizations, all-cause mortality, composite of death/HF hospitalization, device safety at 5 years.	TEER reduced annualized HF hospitalization (33.1 vs. 57.2%, Hazard Ratio (HR) 0.53), lowered all-cause mortality (57.3 vs. 67.2%, HR 0.72), and reduced combined death/HF hospitalization (73.6 vs. 91.5%, HR 0.53) at 5 years. Device-related complications remained rare (1.4%). TF interpretation: landmark long-term data confirming durable benefit of TEER in carefully selected ventricular SMR patients; foundational evidence for the 2025 upgrade of TEER to Class I, LOE A.
Antker, 2024, RESHAPE-HF2, PMID: 39216092	505 patients with HF and moderate–severe functional MR, symptomatic despite guideline-directed medical therapy, from 30 sites in 9 countries. Mean age ≈71 years; LVEF <50%.	TEER (MitraClip) + GDMT vs. GDMT alone.	Three co-primary endpoints: (i) composite of first or recurrent HF hospitalization or cardiovascular (CV) death at 24 months; (ii) first or recurrent HF hospitalization at 24 months; and (iii) change in KCCQ-OS score at 12 months.	At 24 months, TEER plus GDMT reduced the composite of HF hospitalization or CV death vs. GDMT alone (37.0 vs. 58.9 events/100 patient-years; relative risk (RR) 0.64, *P* = 0.002), lowered HF hospitalization (RR 0.59, *P* = 0.002), and improved quality of life at 12 months (KCCQ +21.6 vs. +8.0; Δ + 10.9 points, *P* < 0.001). Device-related complications were infrequent (1.6%). TF interpretation: RESHAPE-HF2 shows TEER provides significant reductions in HF events and QoL improvement in symptomatic HF patients with moderate–severe functional MR on GDMT, supporting its integration into management when anatomy is suitable.
Baldus, 2024, MATTERHORN, PMID:39216093	210 patients with heart failure and secondary (functional) mitral regurgitation who remained symptomatic despite guideline-directed medical therapy. Mean age 70.5 ± 7.9 years, 39.9% women, mean LVEF 43.0 ± 11.7%. (Conducted in Germany; key exclusions per protocol included patients unsuitable for either intervention or with contraindications to randomized treatment.)	TEER (intervention group) vs. mitral-valve surgery (repair or replacement), randomized 1:1.	Primary efficacy: composite at 1 year of death, hospitalization for heart failure, mitral-valve reintervention, implantation of a left-ventricular assist device, or stroke. Primary safety: composite of major adverse events within 30 days after the procedure.	At 1 year, TEER was non-inferior to surgery for the composite efficacy endpoint (16.7 vs. 22.5%). Major adverse events at 30 days were far lower with TEER (14.9 vs. 54.8%). TF interpretation: TEER offers similar 1-year outcomes with markedly better short-term safety, supporting its role as a less-invasive alternative in appropriately selected patients; choice should be individualized.
Tricuspid regurgitation				
Sorajja, 2023, TRILUMINATE, PMID:36876753	350 patients with severe, symptomatic tricuspid regurgitation, mean age 78 years, 54.9% women, enrolled at 65 centres in the US, Canada, and Europe. All were deemed suitable for TEER and optimized on medical therapy.	T-TEER vs. medical therapy alone.	Primary endpoint: hierarchical composite of all-cause death or tricuspid surgery, HF hospitalization, and ≥15-point improvement in KCCQ at 1 year. Secondary: TR severity reduction, KCCQ change, and safety (30-day major adverse events).	Primary endpoint favoured TEER (win ratio 1.48; 95% confidence interval, CI 1.06–2.13; *P* = 0.02). TR reduction was substantial (≤moderate in 87 vs. 4.8% at 30 days; *P* < 0.001). KCCQ improved more with TEER (+12.3 vs. +0.6 points; *P* < 0.001). Death, tricuspid surgery, and HF hospitalization rates were similar between groups. Safety was excellent (98.3% free from major adverse events at 30 days). TF interpretation: T-TEER safely reduces TR severity and substantially improves quality of life, though survival and hospitalization benefits were not demonstrated at 1 year; longer-term outcomes remain under evaluation.
Donal, 2025, Tri-Fr, PMID:39602173	300 patients with severe, symptomatic tricuspid regurgitation despite optimized medical therapy, recruited from 24 centres in France and Belgium (2021–2023). Mean age 78 years, 63.7% women.	T-TEER + OMT vs. OMT alone.	Primary outcome: composite at 1 year of change in NYHA class, change in patient global assessment, or major CV events. Secondary outcomes (hierarchical testing): TR severity, KCCQ score, patient global assessment, composite of all-cause death, tricuspid surgery, KCCQ improvement, or HF hospitalization.	At 1 year, the primary composite was improved in 74.1% of the T-TEER group vs. 40.6% with OMT alone. Severe TR persisted in only 6.8 vs. 53.5% (*P* < 0.001). KCCQ score was higher with T-TEER (69.9 vs. 55.4; *P* < 0.001). Secondary composite favoured T-TEER (win ratio 2.06; 95% CI 1.38–3.08). TF interpretation: T-TEER substantially reduced TR severity and improved patient-reported outcomes compared with medical therapy, supporting its use in symptomatic severe TR, though longer-term follow-up for survival outcomes is needed.
Hahn, 2025, TRISCEND II, PMID:39475399	400 patients with severe, symptomatic tricuspid regurgitation despite medical therapy, enrolled at international centres. Mean age ∼78 years; majority women. Patients had anatomy suitable for transcatheter valve replacement.	TTVR + medical therapy (*n* = 267) vs. medical therapy alone (*n* = 133).	Primary: hierarchical composite of death, RV assist device/heart transplant, postindex valve intervention, HF hospitalization, and improvements in KCCQ-OS (≥10 points), NYHA class (≥1), and 6-minute walk distance (≥30 m). Key safety: severe bleeding, need for new pacemaker.	At 1 year, the primary outcome favoured TTVR (win ratio 2.02; 95% CI 1.56–2.62; *P* < 0.001). Improvements were driven by better quality of life (KCCQ + 23.1 vs. 6.0%), NYHA class, and 6MWD. Mortality and HF hospitalization rates were similar. Safety: higher severe bleeding (15.4 vs. 5.3%) and new pacemaker implantation (17.4 vs. 2.3%) in the TTVR group. TF interpretation: TTVR significantly improved symptoms and quality of life over medical therapy alone, though at the cost of higher procedural risks; long-term data on survival and HF outcomes are awaited.
Gammie, 2022 CTSN, PMID:34767705	401 patients undergoing mitral-valve surgery for severe degenerative MR, with either moderate TR or less-than-moderate TR plus annular dilatation. Randomized intraoperatively.	Mitral-valve surgery + concomitant tricuspid annuloplasty (TA) vs. mitral-valve surgery alone.	Primary 2-year composite: tricuspid reoperation, TR progression by ≥2 grades or to severe TR, or death. Secondary: mortality, TR progression, major adverse cardiac/cerebrovascular events, functional status, QoL, and permanent pacemaker requirement.	At 2 years, the primary endpoint was less frequent with TA (3.9 vs. 10.2%; RR 0.37; *P* = 0.02), mainly due to less TR progression (0.6 vs. 6.1%). Mortality and major adverse events were similar. Permanent pacemaker implantation was higher with TA (14.1 vs. 2.5%). TF interpretation: Concomitant TA during mitral surgery reduced TR progression but increased pacemaker need; long-term follow-up is required to clarify net clinical benefit.

The guidelines also confirm a structured Heart Team-based algorithm (*Table 2*) for choosing between TEER, transcatheter mitral valve replacement (TMVR), and conservative therapy in non-surgical candidates. Based on detailed TOE characterization, cases are classified as: (i) ideal for TEER; (ii) complex for TEER (may require advanced expertise or combination strategies); (iii) very complex for TEER (borderline anatomy, limited chance of durable success); and (iv) unsuitable for TEER [candidates for transcatheter mitral valve implantation (TMVI)].

**Table 2:** Decision-making framework for primary mitral regurgitation: anatomical features for selecting between transcatheter vs. surgical repair/replacement

Ideal for mitral TEER	Suitable for mitral TEER	Challenging for mitral TEER	Mitral TEER impossible
TEER	TEER in experienced centre	TEER in experienced centre	TMVI or Surgery
Central pathology No calcification MVA>4.0 cm^2^ Posterior leaflet >10 mm Tenting height <10 mm Flail gap <10 mm Flail width <15 mm	Isolated commissural lesion (A1/P1 or A3/P3) MAC without leaflet involvement MVA 3.5–4.0 cm^2^ Posterior leaflet length 7–10 mm Tenting height >10 mm Asymmetric tethering Coaptation reserve <3 mm Leaflet-to-anulus index <1.2 Flail width >15 mm Flail gap >10 mm Two jets from leaflet indentations	Commissural lesion with multiple jets MAC with leaflet involvement Fibrotic leaflets Wide jet involving the whole coaptation MVA 3.0–3.5 cm^2^ Posterior leaflet length 5–7 mm Barlow’s disease Cleft Failed surgical annuloplasty	Concentric MAC with stenosis MVA <3.0 cm^2^ Relevant MS (mean gradient >5 mmHg) Posterior leaflet <5 mm Calcification in the grasping zone Deep regurgitate cleft Leaflet perforation Multiple/wide jets Rheumatic MS
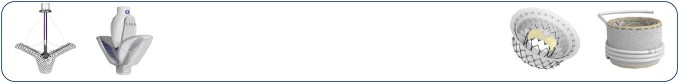			

This pragmatic approach acknowledges the heterogeneity of degenerative MR and aligns treatment strategy with both patient risk factors, anatomical feasibility and patient preference.

Finally, TMVI is acknowledged as an emerging option for anatomies unsuitable for TEER. While early registry data demonstrate feasibility and symptomatic benefit, the guidelines caution about unresolved challenges including high screening failure rates, risk of left ventricular outflow tract obstruction, valve thrombosis, and uncertain durability.^15^

In summary, compared with 2021, the new recommendations on PMR emphasize earlier surgical timing (with LVESDi now incorporated and a new Class I indication for asymptomatic MR), greater confidence in TEER for high-risk patients, and a Heart Team-driven anatomical algorithm that clarifies when TMVR can be considered.

Taken together, these revisions emphasize two important messages: first, the guidelines continue to promote early surgery in asymptomatic PMR, provided durable repair is feasible and surgical risk is low; second, TEER is now recognized as a validated alternative in patients unsuitable for surgery, no longer confined to a marginal recommendation. For clinical practice, this reinforces the need for systematic Heart Team evaluation, incorporating LVESDi and atrial/pulmonary hypertension (PH) markers into surgical timing, and considering TEER more confidently in high-risk subsets.

## Secondary mitral regurgitation

2025 ESC/EACTS guidelines introduce several important refinements in the understanding and management of secondary mitral regurgitation (SMR). Compared with the 2021 version, the current document places stronger emphasis on the distinction between atrial and ventricular SMR as two separate phenotypes, each with different pathophysiology, risk profile, prognosis, and therapeutic implications. Ventricular SMR, the more prevalent and prognostically adverse form, is driven by LV dilatation and systolic dysfunction leading to leaflet tethering and restricted motion.^16^ By contrast, atrial SMR is associated with chronic AF and/or heart failure with preserved ejection fraction (HFpEF), with preserved LV function but annular dilatation and planar leaflet coaptation.^17-19^ Importantly, the guidelines caution that in advanced stages the phenotypes may overlap, underlining the need for careful echocardiographic characterization to avoid misclassification as primary MR.^20^

**Figure 2:**
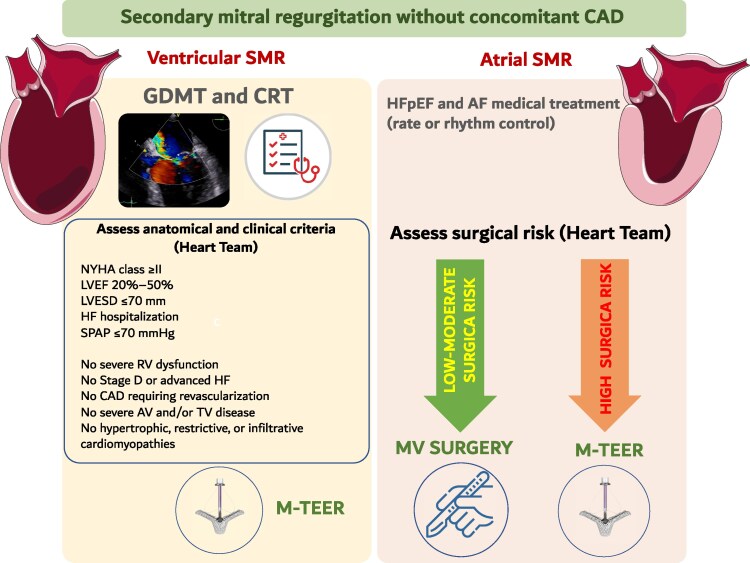
Management algorithm for secondary mitral regurgitation. Adapted from Praz *et al*.^2^

The most significant update concerns the indications for intervention in ventricular SMR. Based on the accumulated evidence from the COAPT (5 years follow-up) and RESHAPE-HF2, as well as supporting meta-analyses, TEER has been upgraded to a Class I, LOE A recommendation in symptomatic patients with severe ventricular SMR, despite guideline-directed medical therapy (GDMT), provided they meet specific criteria. This is a significant step forward compared with the 2021 guidelines, where TEER carried only a Class IIa, LOE B recommendation (*Figure 2*).

By contrast, for atrial SMR, high-quality evidence remains limited, with management largely guided by registry data and expert consensus. Here, the guidelines highlight the importance of treating underlying conditions—AF, HFpEF, —while recognizing the potential role of both surgery and TEER in selected patients, pending stronger trial data.

### Diagnostic assessment

Accurate assessment of SMR requires evaluation under optimized GDMT and in a euvolaemic, normotensive state. Transthoracic echocardiography is the first-line modality, providing assessment of MR severity, LV and LA dimensions, and underlying cardiac pathology. For SMR, lower quantitative thresholds may define severe regurgitation due to elliptical orifice geometry and low-flow states: an EROA ≥ 30 mm^2^ and/or regurgitant volume ≥ 45 mL are associated with adverse outcomes and improved prognosis when corrected.^21^ Transoesophageal echocardiography is indicated for pre-interventional planning, while cardiovascular magnetic resonance (CMR) can confirm severity, quantify chamber volumes, and identify myocardial fibrosis, a key prognostic marker in ventricular SMR.^22^ Exercise echocardiography may reveal dynamic increases in regurgitation and pulmonary pressures in patients with inconclusive findings at rest.

Diagnostic criteria for atrial SMR include preserved LVEF (≥50%) with no regional wall motion abnormalities, no or minimal LV dilatation [LV end-diastolic dimension <56 mm (women) or <63 mm (men)], annular enlargement (anteroposterior diameter >35 mm), and LA dilatation (LAVI >34 mL/m^2^).^23,24^ Clinical context—particularly AF and/or HFpEF—supports this diagnosis. Ventricular SMR is typically identified by reduced LVEF, leaflet tethering with restricted motion and secondary annular dilation.

### Indications for intervention

#### Ventricular secondary mitral regurgitation

The management of ventricular SMR has evolved substantially in the 2025 ESC/EACTS guidelines, with the most important shift being the formal recognition of TEER as a first-line interventional therapy (Class I, LOE A) in appropriately selected patients. This upgrade reflects the strength of recent randomized evidence, which has established TEER as an outcome-modifying treatment for patients who remain symptomatic despite optimized GDMT and cardiac resynchronization therapy (CRT), when indicated. Echocardiographic and clinical criteria, derived from RCTs, must be fulfilled to have a Class I indication for TEER. A Class IIb LOE C is still mantained for patients who do not fulfill optimal criteria with or without advanced heart failure.

The markedly positive results of the COAPT trial, where TEER reduced both heart failure (HF) hospitalizations and all-cause mortality compared with GDMT with durable benefit out to 5 years, played a significant role in the guideline recommendation process.^21,25^ The contrasting neutral results of MITRA-FR^26^ highlighted the critical role of trial design and patient selection.^27^ The recent RESHAPE-HF2 trial further clarified this landscape. By enrolling patients with less severe MR than prior trials and less LV dilatation than those in MITRA-FR, it confirmed that TEER reduces HF hospitalizations and improves quality of life, even in the absence of a definitive mortality benefit (*Table 1*).^28^ A meta-analysis pooling these three RCTs consolidated the message: TEER significantly reduces recurrent HF events and improves clinical status.^29^

In contrast, the case for surgery in isolated ventricular SMR remains far less compelling. Observational series have failed to show a consistent survival advantage with surgical repair or replacement.^30^ The MATTERHORN trial provided a direct comparison, demonstrating that TEER was non-inferior to surgery at 1 year for a composite endpoint of mortality, HF hospitalization, reintervention, left ventricular assist device implantation, or stroke—with the added advantage of a superior safety profile.^31^

Surgery retains a role in ischaemic SMR requiring concomitant coronary artery bypass grafting (CABG), where mitral valve repair or replacement at the time of revascularization remains recommended, provided surgical risk is acceptable. However, the limitations of undersized annuloplasty—with high rates of recurrent MR in patients with marked leaflet tethering or large tenting areas—have been reported. Whether replacement is a better option is still matter of debate.^32,33^

Taken together, these data explain the strong guideline endorsement of TEER in ventricular SMR. In daily practice, the default pathway is now GDMT ± CRT followed by TEER in eligible anatomy, with surgery reserved for patients undergoing CABG or those with valve anatomy unsuitable for transcatheter repair and acceptable surgical risk. The guidelines thus confirm what clinical practice had already begun to anticipate: TEER has moved from a ‘promising option’ to the standard of care in this challenging population.

#### Atrial secondary mitral regurgitation

Compared with ventricular SMR, treatment strategies for atrial SMR remain less clearly defined, mainly due to the lack of dedicated RCTs. Patients are typically elderly and frequently present with AF and marked LA dilatation. Management should begin with optimization of comorbidities, following established guidelines for AF and HFpEF. In particular, Sodium-Glucose cotransporter-2 inhibitors (SGLT2i) are now recommended in HFpEF given their proven prognostic benefit.^34^ Rhythm control strategies may also attenuate atrial SMR severity by reducing LA dilatation and restoring more favourable annular geometry.^35^

A major novelty of the 2025 ESC/EACTS guidelines is the formal recommendation that in symptomatic patients with severe atrial SMR despite optimal medical therapy, mitral valve surgery should be considered in combination with surgical AF ablation (when indicated) and left atrial appendage occlusion (LAAO) (Class IIa, LOE B). This shift reflects accumulating evidence that atrial SMR is not a benign entity and that addressing both valve incompetence and atrial pathology can improve outcomes. Registry data demonstrate that surgical annuloplasty in atrial SMR is associated with durable MR reduction, fewer HF hospitalizations, and improved survival compared with medical therapy alone.^36,37^ A recent meta-analysis further supports the addition of surgical AF ablation, showing reduced arrhythmia recurrence and favourable clinical outcomes.^38^

Transcatheter edge-to-edge repair has also been explored in atrial SMR, mainly through observational studies. These demonstrate feasibility, high procedural success, and symptomatic benefit.^23,39^ Nevertheless, anatomical features such as planar leaflet coaptation, broad central jets, and limited valve area may reduce procedural efficacy.^40^ A small subgroup analysis of the MATTERHORN RCT (*n* = 34) suggested that outcomes with TEER may be comparable to surgery in selected patients, but robust prospective data are lacking.^41^

Taken together, the updated guidelines provide a clearer therapeutic pathway for atrial SMR: (i) optimize comorbidities, particularly AF and HFpEF; (ii) consider surgery with annuloplasty, AF ablation, and LAAO in symptomatic patients despite medical therapy, provided surgical risk is acceptable; and (iii) reserve TEER for high-risk patients or those with suitable anatomy who are not candidates for surgery.

This structured approach represents a significant step forward compared with the 2021 guidelines, which offered little direction for this phenotype. However, larger prospective trials are still needed to define the optimal balance between surgery and transcatheter repair in atrial SMR.

## Tricuspid regurgitation

Tricuspid regurgitation is a frequent echocardiographic finding, especially in elderly patients and women. While trivial or mild TR is usually benign, clinically significant regurgitation (≥ moderate) carries important prognostic implications, being independently associated with HF and excess mortality.^42,43^

Primary TR, due to intrinsic valve pathology, remains rare. The vast majority of patients present with secondary TR, driven by annular dilatation and leaflet tethering in the setting of AF, right atrial (RA) enlargement, PH, or right ventricular (RV) remodelling. Atrial and ventricular mechanisms often overlap, yet current management recommendations continue to follow the simpler primary vs. secondary and associated vs not associated to left-sided valve disease classification.^44^

**Figure 3:**
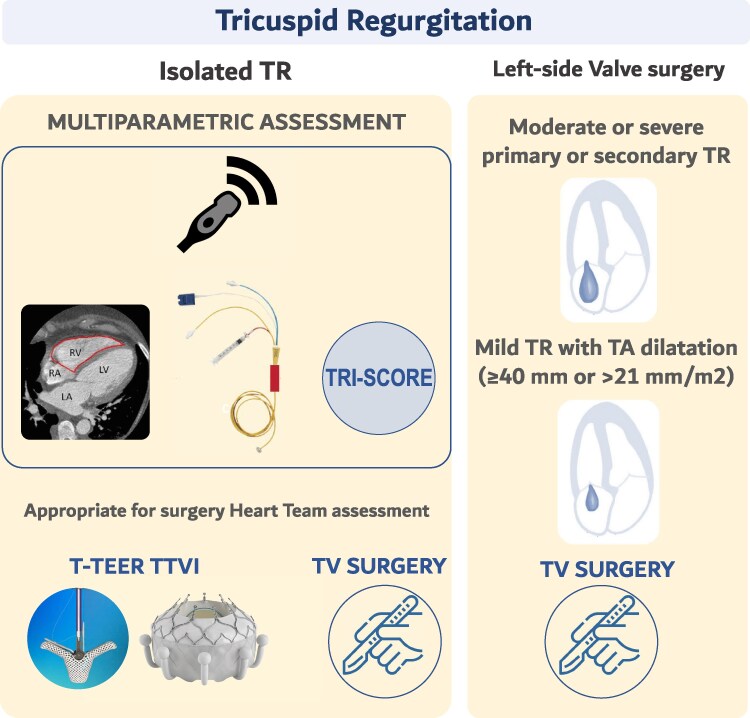
Management algorithm for tricuspid regurgitation. Adapted from Praz *et al*.^2^

2025 ESC/EACTS guidelines introduce two major novelties. First, they emphasize that any intervention decision in severe TR should be preceded by a comprehensive Heart Team evaluation, integrating disease stage (severity of TR, RV and LV function, pulmonary pressures), operative risk, and likelihood of recovery (Class I, LOE C). Second, the role of transcatheter tricuspid intervention has been upgraded (Class IIa, LOE A) in high-risk patients with symptomatic severe TR despite optimal medical therapy, provided RV dysfunction and pre-capillary PH are absent. This reflects the growing body of evidence demonstrating that transcatheter repair or replacement can safely improve symptoms and promote RV reverse remodelling (*Figure 3*).

### Diagnostic assessment

The cornerstone of TR evaluation remains echocardiography. Transthoracic echocardiography is first line, providing assessment of aetiology [primary vs. secondary, including cardiac implantable electronic device (CIED) interaction], grading of regurgitation, and quantification of its impact on right atrial and ventricular size and function. Transoesophageal echocardiography is essential when detailed anatomic visualization is required or for procedural planning.^45^ Severity of TR should be assessed in euvolaemic conditions and by a multiparametric approach. A key modification is the adoption of a five-grade classification (mild, moderate, severe, massive, torrential), which allows better prognostic stratification and more precise evaluation of procedural success.^46^ Importantly, intervention should not be delayed once TR reaches the severe threshold, as progression is associated with increasing symptoms and event risk.^47^

Assessment of RV function remains challenging because of its complex geometry and load dependency, and dysfunction is frequently underestimated in severe TR. Conservative thresholds are advised to detect impairment early, while CMR remains the reference for accurate and reproducible RV volumetry.^48^

Since echocardiography often underestimates pulmonary pressures, right heart catheterization is mandatory before intervention to document RA and RV pressures, venous congestion, and to exclude pre-capillary PH.^49^ Cardiac computed tomography with dedicated right-sided protocols is increasingly used for transcatheter planning, enabling accurate measurement of annular dimensions, coronary anatomy, and vena cava morphology.^44^

Finally, the new guidelines highlight the importance of comprehensive risk stratification. The TRI-SCORE, validated across multicentre cohorts, integrates RV dysfunction and end-organ impairment to identify patients who are likely to benefit from surgery vs. those at high risk of futility.^50,51^ Patients with low-to-intermediate TRI-SCORE (≤5) derive the greatest benefit from early intervention, underscoring the value of systematic Heart Team discussion (*Table 1)*.

### Indications for intervention

Management of clinically relevant TR begins with addressing the underlying cause. This includes guideline-directed treatment for HF, pulmonary vasodilators for PH, and rhythm control in patients with AF.^52^

In patients with HF symptoms, diuretics are the mainstay, typically started with loop diuretics and, when needed, combined with mineralocorticoid receptor antagonists, thiazides, or SGLT2 inhibitors.^53^

While these measures improve congestion and symptoms, they do not alter the natural history of TR. Importantly, medical therapy should not delay timely referral to an expert Heart Valve Centre when intervention is appropriate.^54^

#### Surgical therapy

Patients are often referred late for surgery, when RV dysfunction and systemic organ damage are already present. In this context, isolated tricuspid valve surgery has been associated with high in-hospital mortality rates of 8–10%.^55^ However, contemporary series show better results when patients are referred earlier in the disease process.

Annuloplasty with a prosthetic ring is the preferred approach whenever feasible, particularly in patients with suitable anatomy and low surgical risk.^56^ Valve replacement is usually reserved for advanced cases with severe annular dilatation and leaflet tethering. For CIED-related TR, management may require lead extraction and/or placement of an epicardial lead, which can improve valve function.^57,58^

#### Patients without indication for left-sided surgery

In symptomatic, operable patients with severe primary TR, surgery is recommended. It may also be considered in patients with secondary TR, or in asymptomatic individuals (primary or secondary) showing RV dilatation or early decline in RV function.^59^ Conversely, patients with advanced biventricular dysfunction or severe PH are at excessive surgical risk.^60^

#### Patients undergoing left-sided valve surgery

Severe primary or secondary TR is unlikely to regress after isolated correction of left-sided valve disease, and late tricuspid reoperation carries high mortality.^61^ For this reason, concomitant tricuspid surgery is recommended when severe TR is present at the time of left-sided valve intervention.

Progression of moderate TR or mild TR with annular dilatation is frequent and associated with worse outcomes post-left sided valve surgery.^62,63^ Large retrospective series and two RCTs indicate that adding annuloplasty prevents TR progression and favours RV remodelling, though it has not shown survival benefit and carries an increased risk of pacemaker implantation.^64,65^ This trade-off requires careful patient selection, considering predictors of annular dilatation (e.g. AF, RA size, pulmonary pressures) against the risk of conduction disturbances.^66^

#### Transcatheter therapy

Multiple transcatheter therapies are now available, including TEER, direct annuloplasty, and orthotopic or heterotopic valve replacement. In appropriately selected patients, these approaches reduce TR to moderate or less in over 80% of cases and improve symptoms.^54,67^ The TRILUMINATE pivotal trial confirmed safety and symptomatic benefit of TEER, with improved quality of life as the main driver of the primary endpoint.^54^ At 2 years, fewer HF hospitalizations were seen in the intervention group despite a high crossover rate.^68^ Similar findings were reported in the Tri.Fr trial, where TEER combined with medical therapy was superior to medical therapy alone, particularly for patient-reported outcomes.^69^

The TRISCEND II trial of transcatheter tricuspid valve replacement (TTVR) confirmed significant improvement in symptoms, quality of life, and RV reverse remodelling, but at the cost of higher procedural risks including major bleeding (≈15%) and frequent need for pacemaker implantation (∼25% at 1 year) (*Table 1*).^70^

Cardiac implantable electronic device lead management (repositioning or extraction) may be considered before tricuspid interventions, although the benefit is uncertain and procedural risks are non-negligible.^57^ For recurrent TR after annuloplasty, surgical replacement remains standard, but transcatheter valve-in-ring and valve-in-valve approaches are feasible in selected high-risk patients.^71,72^

Overall, transcatheter therapies represent a rapidly growing option for high-risk patients with symptomatic severe TR. As with surgery, these procedures should be performed in experienced Heart Valve Centres, with careful clinical and anatomical selection to optimize patient outcomes.

## Data Availability

No new data were generated or analyzed in this study. Data sharing is not applicable to this article.
